# Supplementary material for: Pyruvate Dehydrogenase Kinase 1 inhibition mediated oxidative phosphorylation enhancement in cartilage promotes osteoarthritis progression
Source: BMC Musculoskelet Disord. 2023 Jul 20;24:597. doi: 10.1186/s12891-023-06585-6 (PMC10357736; doi:10.1186/s12891-023-06585-6)
Supplement: Supplementary file 1 — Additional file 1. [file 12891_2023_6585_MOESM1_ESM.zip › MF.Gsea.1653627316242/GOMF_L_AMINO_ACID_TRANSMEMBRANE_TRANSPORTER_ACTIVITY.html]

Details for gene set GOMF\_L\_AMINO\_ACID\_TRANSMEMBRANE\_TRANSPORTER\_ACTIVITY[GSEA]

|  || Dataset | 55457\_collapsed\_to\_symbols.cls.cls #OA\_versus\_normal.cls.cls #OA\_versus\_normal\_repos |
| Phenotype | cls.cls#OA\_versus\_normal\_repos |
| Upregulated in class | normal |
| GeneSet | GOMF\_L\_AMINO\_ACID\_TRANSMEMBRANE\_TRANSPORTER\_ACTIVITY |
| Enrichment Score (ES) | -0.42966115 |
| Normalized Enrichment Score (NES) | -1.5853498 |
| Nominal p-value | 0.010204081 |
| FDR q-value | 0.1504283 |
| FWER p-Value | 0.998 |
Table: GSEA Results Summary

  

Fig 1: Enrichment plot: GOMF\_L\_AMINO\_ACID\_TRANSMEMBRANE\_TRANSPORTER\_ACTIVITY      
 Profile of the Running ES Score & Positions of GeneSet Members on the Rank Ordered List

  

| SYMBOL | TITLE | RANK IN GENE LIST | RANK METRIC SCORE | RUNNING ES | CORE ENRICHMENT || 1 | SLC7A7 | solute carrier family 7 member 7 [Source:HGNC Symbol;Acc:HGNC:11065] | 1051 | 0.506 | -0.0420 | No |
| 2 | SLC1A4 | solute carrier family 1 member 4 [Source:HGNC Symbol;Acc:HGNC:10942] | 1372 | 0.447 | -0.0317 | No |
| 3 | SLC3A1 | solute carrier family 3 member 1 [Source:HGNC Symbol;Acc:HGNC:11025] | 2706 | 0.279 | -0.1134 | No |
| 4 | SLC38A6 | solute carrier family 38 member 6 [Source:HGNC Symbol;Acc:HGNC:19863] | 3060 | 0.244 | -0.1217 | No |
| 5 | CTNS | "cystinosin, lysosomal cystine transporter [Source:HGNC Symbol;Acc:HGNC:2518]" | 3337 | 0.218 | -0.1261 | No |
| 6 | SLC43A1 | solute carrier family 43 member 1 [Source:HGNC Symbol;Acc:HGNC:9225] | 3384 | 0.213 | -0.1129 | No |
| 7 | SLC22A2 | solute carrier family 22 member 2 [Source:HGNC Symbol;Acc:HGNC:10966] | 4435 | 0.118 | -0.1853 | No |
| 8 | SLC25A12 | solute carrier family 25 member 12 [Source:HGNC Symbol;Acc:HGNC:10982] | 5506 | 0.032 | -0.2660 | No |
| 9 | SLC38A7 | solute carrier family 38 member 7 [Source:HGNC Symbol;Acc:HGNC:25582] | 5916 | 0.001 | -0.2977 | No |
| 10 | SLC25A15 | solute carrier family 25 member 15 [Source:HGNC Symbol;Acc:HGNC:10985] | 6086 | -0.011 | -0.3100 | No |
| 11 | SLC7A11 | solute carrier family 7 member 11 [Source:HGNC Symbol;Acc:HGNC:11059] | 6470 | -0.040 | -0.3366 | No |
| 12 | SLC17A7 | solute carrier family 17 member 7 [Source:HGNC Symbol;Acc:HGNC:16704] | 6773 | -0.061 | -0.3553 | No |
| 13 | SLC1A2 | solute carrier family 1 member 2 [Source:HGNC Symbol;Acc:HGNC:10940] | 7026 | -0.082 | -0.3685 | No |
| 14 | SLC6A5 | solute carrier family 6 member 5 [Source:HGNC Symbol;Acc:HGNC:11051] | 7439 | -0.114 | -0.3916 | No |
| 15 | SLC36A1 | solute carrier family 36 member 1 [Source:HGNC Symbol;Acc:HGNC:18761] | 7670 | -0.132 | -0.3991 | No |
| 16 | OCA2 | OCA2 melanosomal transmembrane protein [Source:HGNC Symbol;Acc:HGNC:8101] | 8064 | -0.160 | -0.4171 | Yes |
| 17 | SLC17A6 | solute carrier family 17 member 6 [Source:HGNC Symbol;Acc:HGNC:16703] | 8119 | -0.164 | -0.4084 | Yes |
| 18 | SLC1A1 | solute carrier family 1 member 1 [Source:HGNC Symbol;Acc:HGNC:10939] | 8157 | -0.167 | -0.3981 | Yes |
| 19 | SLC7A10 | solute carrier family 7 member 10 [Source:HGNC Symbol;Acc:HGNC:11058] | 8492 | -0.193 | -0.4089 | Yes |
| 20 | SLC1A7 | solute carrier family 1 member 7 [Source:HGNC Symbol;Acc:HGNC:10945] | 8504 | -0.194 | -0.3945 | Yes |
| 21 | SLC7A2 | solute carrier family 7 member 2 [Source:HGNC Symbol;Acc:HGNC:11060] | 8518 | -0.195 | -0.3802 | Yes |
| 22 | SLC25A22 | solute carrier family 25 member 22 [Source:HGNC Symbol;Acc:HGNC:19954] | 8682 | -0.208 | -0.3766 | Yes |
| 23 | SLC6A9 | solute carrier family 6 member 9 [Source:HGNC Symbol;Acc:HGNC:11056] | 8704 | -0.209 | -0.3618 | Yes |
| 24 | SLC25A13 | solute carrier family 25 member 13 [Source:HGNC Symbol;Acc:HGNC:10983] | 8713 | -0.210 | -0.3459 | Yes |
| 25 | SLC7A9 | solute carrier family 7 member 9 [Source:HGNC Symbol;Acc:HGNC:11067] | 9539 | -0.277 | -0.3883 | Yes |
| 26 | SLC1A3 | solute carrier family 1 member 3 [Source:HGNC Symbol;Acc:HGNC:10941] | 9656 | -0.288 | -0.3747 | Yes |
| 27 | SLC25A38 | solute carrier family 25 member 38 [Source:HGNC Symbol;Acc:HGNC:26054] | 9904 | -0.312 | -0.3693 | Yes |
| 28 | SLC7A1 | solute carrier family 7 member 1 [Source:HGNC Symbol;Acc:HGNC:11057] | 10131 | -0.334 | -0.3607 | Yes |
| 29 | SLC6A7 | solute carrier family 6 member 7 [Source:HGNC Symbol;Acc:HGNC:11054] | 10185 | -0.339 | -0.3382 | Yes |
| 30 | SLC7A8 | solute carrier family 7 member 8 [Source:HGNC Symbol;Acc:HGNC:11066] | 10228 | -0.344 | -0.3144 | Yes |
| 31 | SERINC5 | serine incorporator 5 [Source:HGNC Symbol;Acc:HGNC:18825] | 10352 | -0.356 | -0.2960 | Yes |
| 32 | SLC47A1 | solute carrier family 47 member 1 [Source:HGNC Symbol;Acc:HGNC:25588] | 10423 | -0.363 | -0.2729 | Yes |
| 33 | SLC38A3 | solute carrier family 38 member 3 [Source:HGNC Symbol;Acc:HGNC:18044] | 10443 | -0.366 | -0.2456 | Yes |
| 34 | SLC38A1 | solute carrier family 38 member 1 [Source:HGNC Symbol;Acc:HGNC:13447] | 10617 | -0.385 | -0.2288 | Yes |
| 35 | SLC1A6 | solute carrier family 1 member 6 [Source:HGNC Symbol;Acc:HGNC:10944] | 10626 | -0.386 | -0.1991 | Yes |
| 36 | SFXN1 | sideroflexin 1 [Source:HGNC Symbol;Acc:HGNC:16085] | 10880 | -0.414 | -0.1863 | Yes |
| 37 | SLC6A20 | solute carrier family 6 member 20 [Source:HGNC Symbol;Acc:HGNC:30927] | 10982 | -0.428 | -0.1605 | Yes |
| 38 | SERINC3 | serine incorporator 3 [Source:HGNC Symbol;Acc:HGNC:11699] | 11087 | -0.442 | -0.1339 | Yes |
| 39 | SLC38A2 | solute carrier family 38 member 2 [Source:HGNC Symbol;Acc:HGNC:13448] | 11722 | -0.536 | -0.1411 | Yes |
| 40 | SLC7A5 | solute carrier family 7 member 5 [Source:HGNC Symbol;Acc:HGNC:11063] | 12203 | -0.646 | -0.1276 | Yes |
| 41 | SLC1A5 | solute carrier family 1 member 5 [Source:HGNC Symbol;Acc:HGNC:10943] | 12501 | -0.748 | -0.0919 | Yes |
| 42 | SLC7A6 | solute carrier family 7 member 6 [Source:HGNC Symbol;Acc:HGNC:11064] | 12551 | -0.774 | -0.0349 | Yes |
| 43 | SLC3A2 | solute carrier family 3 member 2 [Source:HGNC Symbol;Acc:HGNC:11026] | 12587 | -0.789 | 0.0243 | Yes |
Table: GSEA details [plain text format]

  

Fig 2: GOMF\_L\_AMINO\_ACID\_TRANSMEMBRANE\_TRANSPORTER\_ACTIVITY      
 Blue-Pink O' Gram in the Space of the Analyzed GeneSet

  

Fig 3: GOMF\_L\_AMINO\_ACID\_TRANSMEMBRANE\_TRANSPORTER\_ACTIVITY: Random ES distribution      
 Gene set null distribution of ES for **GOMF\_L\_AMINO\_ACID\_TRANSMEMBRANE\_TRANSPORTER\_ACTIVITY**
